# Neural connectivity between the hypothalamic supramammillary nucleus and appetite‐ and motivation‐related regions of the rat brain

**DOI:** 10.1111/jne.12829

**Published:** 2020-01-29

**Authors:** Fabrice Plaisier, Catherine Hume, John Menzies

**Affiliations:** ^1^ Centre for Discovery Brain Sciences Edinburgh Medical School: Biomedical Sciences University of Edinburgh Edinburgh UK; ^2^ ZJU‐UoE Institute Zhejiang University School of Medicine, Zhejiang University International Campus Haining Zhejiang China

**Keywords:** food, motivation, neuroanatomy, supramammillary nucleus, tracing

## Abstract

The supramammillary nucleus (SuM) has an emerging role in appetite control. We have shown that the rat SuM is activated during hunger or food anticipation, or by ghrelin administration. In the present study, we characterised the connectivity between the SuM and key appetite‐ and motivation‐related nuclei in the rat. In adult wild‐type rats, or rats expressing Cre recombinase under the control of the tyrosine hydroxylase (TH) promoter (TH‐Cre rats), we used c‐Fos immunohistochemistry to visualise and correlate the activation of medial SuM (SuMM) with activation in the lateral hypothalamic area (LH), the dorsomedial hypothalamus (DMH) or the ventral tegmental area (VTA) after voluntary consumption of a high‐sugar, high‐fat food. To determine neuroanatomical connectivity, we used retrograde and anterograde tracing methods to specifically investigate the neuronal inputs and outputs of the SuMM. After consumption of the food there were positive correlations between c‐Fos expression in the SuMM and the LH, DMH and VTA (*P* = 0.0001, 0.01 and 0.004). Using Fluoro‐Ruby as a retrograde tracer, we demonstrate the existence of inputs from the LH, DMH, VTA and ventromedial hypothalamus (VMH) to the SuMM. The SuMM showed reciprocal inputs to the LH and DMH, and we identified a TH‐positive output from SuMM to DMH. We co‐labelled retrogradely‐labelled sections for TH in the VMH, or for TH, orexin and melanin‐concentrating hormone in the LH and DMH. However, we did not observe any colocalisation of immunoreactivity with any retrogradely‐labelled cells. Viral mapping in TH‐Cre rats confirms the existence of a reciprocal SuMM‐DMH connection and shows that TH‐positive cells project from the SuMM and VTA to the lateral septal area and cingulate cortex, respectively. These data provide evidence for the connectivity of the SuMM to brain regions involved in appetite control, and form the foundation for functional and behavioural studies aiming to further characterise the brain circuitry controlling eating behaviours.

## 1 INTRODUCTION

A picture is gradually emerging of the functional and anatomical connectivity between the neuroanatomically defined brain regions involved in appetite control. Understanding these circuits is currently one of the most challenging barriers to the development of treatment options for eating disorders and obesity. In the present study, we set out to examine the connectivity of the hypothalamic supramammillary nucleus (SuM), a region with an emerging role in metabolic control. We have previously shown that SuM Fos expression is increased with fasting and food anticipation, that peripheral administration of the orexigenic hormone ghrelin increases SuM Fos expression and also evokes an excitatory response in single SuM cells recorded electrophysiologically in vivo, and that intra‐SuM ghrelin injection induces feeding in rats.^1^ In addition, an anorexigenic glucagon‐like peptide 1 (GLP‐1)‐oestrogen conjugate molecule^2^ or a GLP‐1 receptor agonist^3^ have been shown to act at the SuM to reduce body weight and motivation for food reward. Pharmacological studies have demonstrated that the SuM mediates reinforcement,^4^, ^5^ revealing a potential role for the SuM in motivated behaviours.

The lateral SuM (SuML) appears to play a role in the generation and maintenance of hippocampal theta rhythm, and the medial SuM (SuMM) contains tyrosine hydroxylase (TH)‐positive neurones that project to areas involved in motivated behaviours such as the lateral septal nucleus and lateral hypothalamic area.^6^, ^7^ Classic tracing studies have deciphered the connectivity of the SuM with respect to its role in memory; however, the potential connectivity of the SuM with reward and appetite‐associated regions has not yet been investigated systematically.

To understand the potential role of the SuMM in motivated feeding behaviours, we first used Fos expression as an indirect marker of neural activation. We determined the effects of consumption of a high‐sugar food on the activity of the SuMM, with focus on TH‐expressing SuMM neurones, and sought correlations between Fos expression in the SuMM and in specific appetite and reward‐associated brain regions. We then used retrograde and anterograde tracers, and Cre‐dependent viral vector‐assisted mapping to trace the afferent and efferent connections of SuMM neurones with these feeding behaviour‐associated brain regions.

## 2 MATERIALS AND METHODS

### 2.1 Animals

Adult wild‐type Sprague‐Dawley rats or Long‐Evans rats expressing Cre recombinase under the TH promoter^8^ (“TH‐Cre rats”; Tg(TH‐Cre)3.1Deis; provided by Dr Karl Deisseroth via the NIH RRRC, RRRC#: 659, Columbia, MO, USA) were single housed under a 12:12 hour light/dark photocycle (lights on at 7.00 am) at 20 ± 1°C with ad lib. access to water and standard diet (RM1; Special Diet Services, Witham, UK). All behavioural and surgical procedures were carried out under UK Home Office regulations after approval from the local ethical committee.

### 2.2 Sweetened condensed milk scheduled‐feeding and Fos immunohistochemistry in the SuMM

Adult Sprague‐Dawley rats (aged 8‐10 weeks) were schedule‐fed a high‐sugar food (sweetened condensed milk [SCM]). For 8 days, SCM (5 mL; diluted 50% v/v in water; 73 kJ; Nestlé, Vevey, Switzerland) was presented in the rat's home cage for 15 minutes each day at the same time each day (either 2.00 or 3.00 pm; “SCM group”, n = 8). Standard diet was available at all times. A separate group of rats with ad lib. access to standard diet, but no SCM access, served as controls (n = 8). Body weights and energy intake from SCM and standard diet were measured in both groups over the 8‐day scheduled feeding paradigm. One hour following the end of SCM access on day 8, rats were given an i.p. overdose of sodium pentobarbital and transcardially perfused with ice‐cold saline containing 0.012% w/v heparin and 4% w/v paraformaldehyde in 0.1 mol L^‐1^ phosphate buffer.

Brains were processed for Fos and TH‐like immunoreactivity, imaged and quantified as described previously.^9^ To detect Fos‐like immunoreactivity, we used anti‐Fos rabbit primary antibody (dilution 1:100 000; 226 003; Synaptic Systems, Göttingen, Germany) and a biotinylated horse anti‐rabbit immunoglobulin (Ig)G secondary antibody (dilution 1:500; BA‐1100; Vector Laboratories, Burlingame, CA, USA). To detect TH‐like immunoreactivity, we used anti‐tyrosine hydroxylase (dilution 1:20 000; MAB318; Merck Millipore, Billerica, MA, USA) mouse primary antibody and a biotinylated horse anti‐mouse IgG secondary antibody (dilution 1:500; BA‐2000; Vector Laboratories).

As negative controls, for the anti‐Fos and anti‐TH primary antibodies, we incubated the antibodies with pre‐immunised rabbit (for Fos) or mouse (for TH) serum. Brain sections stained with these incubated antibodies showed no Fos‐ or TH‐like immunoreactivity. For the anti‐Fos primary antibody, we also carried out positive and pre‐absorption controls. Positive Fos control brain sections were generated by giving rats a hyperosmotic stimulus (3.5 mol L^‐1^ NaCl. 600 µL kg^‐1^, administered i.p.) and processing brain sections as described.^9^ Dense Fos+ staining was observed in osmosensitive brain regions, including the supraoptic and paraventricular nucleus of the hypothalamus. The Fos antibody was preabsorbed by incubation overnight with an excess of purified Fos peptide (226‐0P; Synaptic Systems). Brain sections from these osmotically challenged rats incubated with the preabsorbed antibody showed no Fos‐like immunoreactivity.

For Fos counting, the same regions in the SuMM, dorsomedial hypothalamus (DMH), lateral hypothalamic area (LHA) and ventral tegmental area (VTA) were counted in each brain. SuMM: between bregma −4.36 and −4.68 mm (three or four sections per brain); DMH: between bregma −3.12 and −3.36 mm (two sections per brain), LHA: between bregma −1.80 and 2.16 mm (three sections per brain) and VTA: between bregma −4.68 and −4.92 mm (two sections per brain). For counting Fos in SuMM TH cells, sections between bregma −4.44 and −4.68 mm (two or three sections per brain) were used. The mean number of Fos+ nuclei per brain section or percentage of Fos+ TH cells was calculated for each rat and group means calculated. Each brain region was counted under blinded conditions by an experienced experimenter. Two rats did not eat SCM on day 8 and were excluded from analysis. Statistical analyses were performed using prism, version 6 (GraphPad Software Inc., San Diego, CA, USA). Differences in Fos expression between groups were determined using Mann‐Whitney tests. *P* < 0.05 was considered statistically significant. Linear regression analysis was carried out to assess the relationship between Fos expression in the SuMM and other brain regions.

### 2.3 Stereotactic injections of tracers

All stereotaxic injections were made using glass micropipettes (tip diameter 10‐15 µm; Drummond Scientific Company, Broomall, PA, USA). Male TH‐Cre rats (7‐9 weeks old, body weight 300 g ± 25 g) were anaesthetised with isoflurane, fixed in a stereotaxic frame, craniotomised and the pipette inserted into the target brain region with reference to bregma and lambda. Following the postoperative intervals appropriate for each tracer, rats were perfused transcardially as above and the brains removed and processed for immunofluorescence. Only rats with injection sites entirely restricted to areas of interest were analysed.

### 2.4 Fluoro‐Ruby retrograde tracing

Fluoro‐Ruby (FR; tetramethylrhodamine‐dextran‐amine; 10% in phosphate buffered saline [PBS]; Life Technologies, Grand Island, NY, USA) was injected by iontophoresis using a current source (Stoelting Co., Wood Dale, IL, USA) at the coordinates (mm) with reference to the bregma^10^: LHA: anteroposterior (AP) −2.5, mediolateral (ML) −1.7, dorsoventral (DV) −9; DMH: AP −3, ML −0.5, DV −8.5; SuM: AP −4.4, ML −0.2, DV −8.4. The current (AC, 7 seconds on, 7 seconds off) was set to +2 µA for 6 minutes for the DMH (n = 5), 8 minutes for the SuMM (n = 10) and 10 minutes for the LHA (n = 7). The pipette was left in place for 15 minutes following each injection. During penetration and withdrawal of the pipette, the current was reversed (−5 µA) to minimise leakage of the tracer along the penetration track. The rats were then left for 21 days for retrograde transport of the tracer. Because rhodamine derivatives exhibit the potential to label non‐neuronal perivascular cells,^11^ a neurone‐specific marker (NeuN) was used to confirm the neuronal phenotype of the FR‐labelled cells.^12^

Free‐floating sections were incubated in 1% sodium borohydride (NaBH_4_; in PBS; Sigma‐Aldrich, St Louis, MO, USA).^13^ Sections were mounted onto slides (VWR International Ltd, Lutterworth, UK) and air‐dried. After blocking (3% goat serum + 0.4% Triton X‐100 in PBS), slides were incubated with anti‐NeuN and either with anti‐TH (dilution 1:1000), anti‐Orexin A (ORX) or anti‐ melanin‐concentrating hormone (MCH) primary antibodies. After washing, slides were incubated with Alexa‐488 and Alexa‐647 dye‐conjugated secondary antibodies. Nuclear DNA was stained with Hoechst 33342 (10 μg mL^‐1^ in PBS; Molecular Probes, Carlsbad, CA, USA) to visualise nuclei. Slides were coverslipped using Fluoromount Medium (Sigma‐Aldrich). No signal was detected after applying secondary antibodies in the absence of primary antibodies. Table 1 provides details of all the antibodies that were used in the present study.

**Table 1:** Primary and secondary antibodies used in the immunofluorescence assays

	Catalogue no.	RRID	Supplier	Dilution	Raised in
Primary Abs					
Fos	226003	http://scicrunch.org/resolver/AB_2231974	Synaptic Systems (Göttingen, Germany)	1:100 000	Rabbit
Green fluorescent protein/yellow fluorescent protein	ab13970	http://scicrunch.org/resolver/AB_300798	Abcam (Cambridge, MA, USA)	1:10 000 or 1:2000	Chicken
HuC/HuD	A‐21271	http://scicrunch.org/resolver/AB_221448	Thermo Fisher Scientific (Waltham, MA, USA)	1:35	Mouse
Melanin‐concentrating hormone	H‐070‐47	http://scicrunch.org/resolver/AB_2722682	Phoenix Pharmaceuticals	1:2500	Rabbit
NeuN	MAB377	http://scicrunch.org/resolver/AB_2298772	Merck Millipore (Billerica, MA, USA)	1:1500	Mouse
Orexin A	H‐003‐30	http://scicrunch.org/resolver/AB_2315019	Phoenix Pharmaceuticals (Belmont, CA, USA)	1:2500	Rabbit
Tyrosine hydroxylase	AB152	http://scicrunch.org/resolver/AB_390204	Merck Millipore	1:1000 or 1:2000	Rabbit
Secondary Abs					
Biotinylated anti‐rabbit immunoglobulin G	BA‐1100	http://scicrunch.org/resolver/AB_2336201	Vector Laboratories (Burlingame, CA, USA)	1:500	Horse
Biotinylated anti‐mouse immunoglobulin G	BA‐2000	http://scicrunch.org/resolver/AB_2313581	Vector Laboratories	1:500	Horse
Alexa Fluor^®^ 488 Anti‐chicken	A‐11039	http://scicrunch.org/resolver/AB_142924	Life Technologies (Grand Island, NY, USA)	1:750	Goat
Alexa Fluor^®^ 488 Anti‐rabbit	A‐11008	http://scicrunch.org/resolver/AB_143165	Life Technologies	1:750	Goat
Alexa Fluor^®^ 546 Anti‐mouse	A‐11030	http://scicrunch.org/resolver/AB_2534089	Life Technologies	1:750	Goat
Alexa Fluor^®^ 546 Anti‐rabbit	A‐11035	http://scicrunch.org/resolver/AB_2534093	Life Technologies	1:750	Goat
Alexa Fluor^®^ 647 Anti‐mouse	A‐21235	http://scicrunch.org/resolver/AB_2535804	Life Technologies	1:750	Goat
Alexa Fluor^®^ 647 Anti‐rabbit	A‐21244	http://scicrunch.org/resolver/AB_2535812	Life Technologies	1:750	Goat

### 2.5 Adenoassociated virus (AAV) anterograde tracing

Male TH‐Cre rats were transfected with AAV to evaluate the potential reciprocal projections from DMH and SuM neurones. We used a Cre‐inducible recombinant AAV viral vector AAVDJ‐EF1a‐DIO‐hChR2(H134R)‐EYFP‐WPRE‐pA (titre: 5.4 × 10^12^ particles/mL; University of North Carolina Vector Core, Chapel Hill, NC, USA) to deliver yellow fluorescent protein (YFP) specifically to TH‐expressing neurones.^8^ The injections were performed unilaterally by pressure injection (50 nL min^‐1^) into the DMH (n = 12) or SuMM (n = 12) according to the coordinates given above. The needle was slowly retracted 10 minutes after injection. Rats were left for 8 weeks for viral expression and protein transport to distal terminals.

To quantify fluorescently labelled TH‐positive neurones at the injection site, SuM sections were immunostained as described above using anti‐GFP/YFP (dilution 1:10 000), anti‐TH (dilution 1:1000) and anti‐NeuN primary antibodies. The Alexa‐488, Alexa‐546 and Alexa‐647 dye‐conjugated secondary antibodies were used. The anti‐GFP antibody was used to amplify the viral‐mediated expression of eYFP signal, especially at the level of the axonal fibres.

Free‐floating tissue sections were washed and subjected to heat‐induced epitope retrieval using 10 mmol L^‐1^ sodium citrate (pH 6) for 3 hours at 90°C. The sections were blocked (10% goat serum + 0.4% Triton X‐100 diluted in PBS) then incubated overnight in anti‐GFP/YFP (dilution 1:2000), anti‐HuC/HuD and anti‐TH (dilution 1:2000) primary antibodies. After washing, sections were incubated in Alexa‐488, Alexa‐546 and Alexa‐647 dye‐conjugated secondary antibodies. Slides were counterstained and mounted as described above. The neuronal RNA‐binding protein HuC/D antibody was used to label the neuronal cell nuclei and cytoplasm.^14^ Contacts between YFP+ labelled fibres and HuC/D immunoreactive neurones were examined by confocal microscopy.

### 2.6 Imaging and image analysis

Nuclear counterstaining and a rat brain atlas^10^ were used to identify the injections sites and labelled regions. Immunoreactivity was visualised with a Nikon A1R confocal microscope (Nikon UK Ltd, Surbiton, UK) using 20× Plan Apo VC/NA 0.8 and 40× Plan Flour/NA 1.3 oil objectives. Confocal images were acquired using line by line sequential scanning with laser excitation wavelengths of 488 nm for GFP/YFP, 561 nm for Alexa‐546 and FR, and 647 nm for Alexa‐647. Standard photomultiplier tubes were used. A XY pixel size of 0.6 µm/0.32 µm and a Z‐step size of 1 µm/0.5 µm were used for the 20× and 40× objectives, respectively. nis‐elements (Nikon UK Ltd) was used for image acquisition. imagej (NIH, Bethesda, MD, USA) was used to produce the maximum intensity Z‐projections and for cell counting. AAV expression was quantified by counting co‐labelled (TH‐positive/YFP+/NeuN+) neurones and (YFP+/NeuN+)‐only labelled neurones. FR‐labelled neurones or YFP+ fibres in the same focal plane and showing similar morphology, position and orientation under the two filters for the detection of FR or YFP and MCH‐, ORX‐ or TH‐immunoreactivity were considered to be double‐labelled. The analysis of YFP+ fibres appositions on HuC/D‐immunoreactive neurones were processed and analysed with imaris, version 9.2.0 (Bitplane AG, Zurich, Switzerland) software. Appositions were confirmed by investigating single optical sections in three planes. The red channel was recoloured to magenta^15^; an overlay of green and magenta appears white.

## 3 RESULTS

### 3.1 Expression of Fos immunoreactivity in the SuMM and appetite‐ and motivation‐related areas in response to the voluntary consumption of SCM

**Figure 1:**
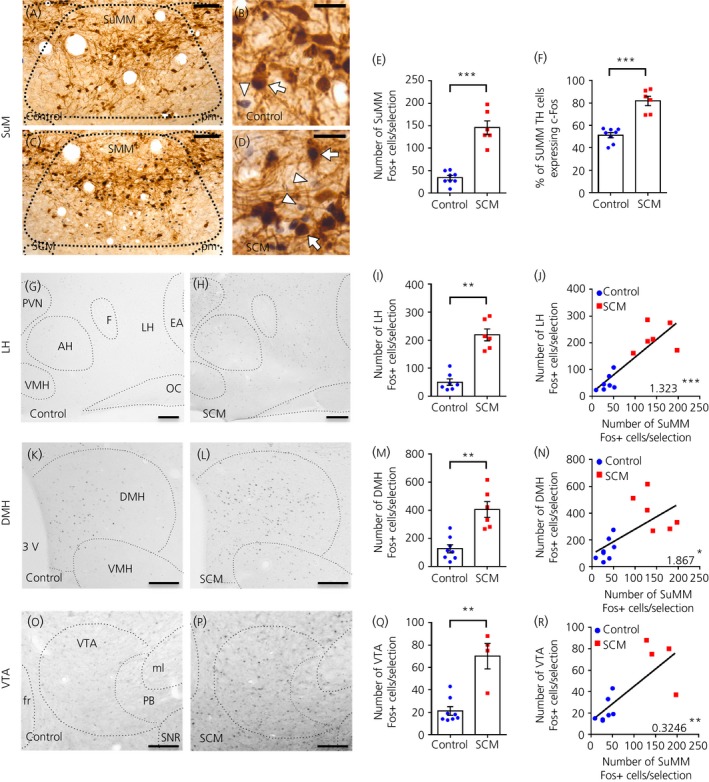
Expression of Fos immunoreactivity in the medial supramammillary nucleus (SuMM) and appetite‐reward related areas in response to the voluntary consumption of sweetened condensed milk (SCM). Data are the mean ± SEM. **P < 0.01, ***P < 0.001. A‐D, Representative images of Fos (arrowheads) and tyrosine hydroxylase (TH) (arrows) immunohistochemistry within SuMM in control (A, B) and SCM‐fed rats (C, D) in coronal sections at bregma −4.5 mm. E, Number of cells expressing Fos per brain section in SuMM for control (n = 8) and SCM (n = 6) groups (*P* = 0.0007, Mann‐Whitney test). F, Percentage of SuMM TH‐immunoreactive cells expressing Fos‐positive nuclei per brain section for control (n = 8) and SCM (n = 6) groups (*P* = 0.0007, Mann‐Whitney test). G and H, Representative images of Fos expression in the lateral hypothalamic area (LH) (bregma −1.72 mm), dorsomedial nucleus of the hypothalamus (DMH) (K, L; bregma −3 mm) and ventral tegmental area (VTA) (O, P; bregma −5.04 mm) in control and SCM‐fed rats. I, M, and Q, Number of cells expressing Fos per brain section for control and SCM groups in the LHA (I; control n = 7, SCM n = 6; *P* = 0.0012, Mann‐Whitney test), DMH (M; control n = 8, SCM n = 6; *P* = 0.0013, Mann‐Whitney test) and VTA (Q; control n = 8, SCM n = 4; *P* = 0.0081, Mann‐Whitney test). J, N, and R, Linear regression plots and coefficients (with significance indicated as *P ≤ 0.05, **P ≤ 0.01 or ***P ≤ 0.001) showing the correlation between Fos expression in the SuMM and Fos expression in the LHA (J; *r* ^2^ = 0.7562, *P* = 0.0001), DMH (N; *r* ^2^ = 0.434, *P* = 0.0104) and VTA (R; *r* ^2^ = 0.5827, *P* = 0.0039). Scale bars represent 200 µm (G, H, K, L, O, P), 50 µm (A, C) and 25 µm (B, D). 3V, third ventricle; AH, anterior hypothalamic nucleus; EA, sublenticular extended amygdala; f, fornix; fr, fasciculus retroflexus; LH, lateral hypothalamic area; ml, medial lemniscus; OC, optic tract; PB, parabrachial pigmented nucleus; pm, principal mammillary tract; PVN, paraventricular nucleus; SNR, substantia nigra; SuM, supramammillary nucleus; VMH, ventromedial nucleus

In line with our previous observations,^16^ there was no divergence in body weight (*P* = 0.63, Mann‐Whitney test) or total daily energy intake (*P* = 0.11, Mann‐Whitney test) between the SCM group and controls during the SCM scheduled‐feeding period (see Supporting information, Figure S1). In the SuMM, Fos expression was significantly higher in the SCM group compared to controls (control, 34 ± 5 nuclei per section; SCM, 146 ± 15 nuclei per section; *P* = 0.0007) (Figure 1A‐E). The percentage of SuMM TH‐positive neurones expressing Fos was significantly higher in brains from rats in the SCM group compared to controls (control, 51.2 ± 2.0% nuclei per section; SCM, 81.7 ± 4.2% nuclei per section; *P* = 0.0007) (Figure 1F). In the LHA, DMH and VTA Fos expression was also significantly higher in the SCM group (LHA: control, 50 ± 12 nuclei per section; SCM, 219 ± 21 nuclei per section; *P* = 0.0012; DMH: control, 126 ± 29 nuclei per section; SCM, 406 ± 57 nuclei per section; *P* = 0.0013; VTA: control, 21 ± 4 nuclei per section; SCM, 70 ± 11 nuclei per section; *P* = 0.0081) (Figure 1G‐I, K‐M, O‐Q). Across control and SCM groups, there was a positive correlation between Fos expression in the SuMM and Fos expression in the LHA, DMH or VTA (LHA: *r* ^2^ = 0.7562, *P* = 0.0001; DMH: *r* ^2^ = 0.434, *P* = 0.0104; VTA: *r* ^2^ = 0.5827, *P* = 0.0039) (Figure 1J,N,R).

In a separate experiment, we gave rats access to SCM only once (“novel” access) and measured Fos expression in the SuMM. Novel access increased Fos expression compared to controls, regardless of whether rats ate the SCM or not (control, 92 ± 10 nuclei per section; consuming SCM, 165 ± 13 nuclei per section; not consuming SCM, 170 ± 16 nuclei per section *P* = 0.0007), although this increase was not observed in the cells that were TH+.

To determine whether Fos expression was related to anticipation of scheduled SCM access, we also measured SuMM Fos expression in rats that were schedule‐fed SCM for 6 days and then, on day 7, rats were presented with SCM at an ‘unexpected’ time (4 hours prior to scheduled access). Fos expression was increased compared to controls (34 ± 5 nuclei per section in rats that had never had SCM access), although there was no difference in Fos expression or percentage of TH+ cells expressing Fos between rats that received SCM at the expected or unexpected time (expected, 146 ± 15 nuclei per section, 60 ± 5% of TH+ cells were Fos+; unexpected, 191 ± 26 nuclei per section; 61 ± 4% of TH+ cells were Fos+).

### 3.2 The SuMM receives inputs from appetite‐ and motivation‐related areas

**Figure 2:**
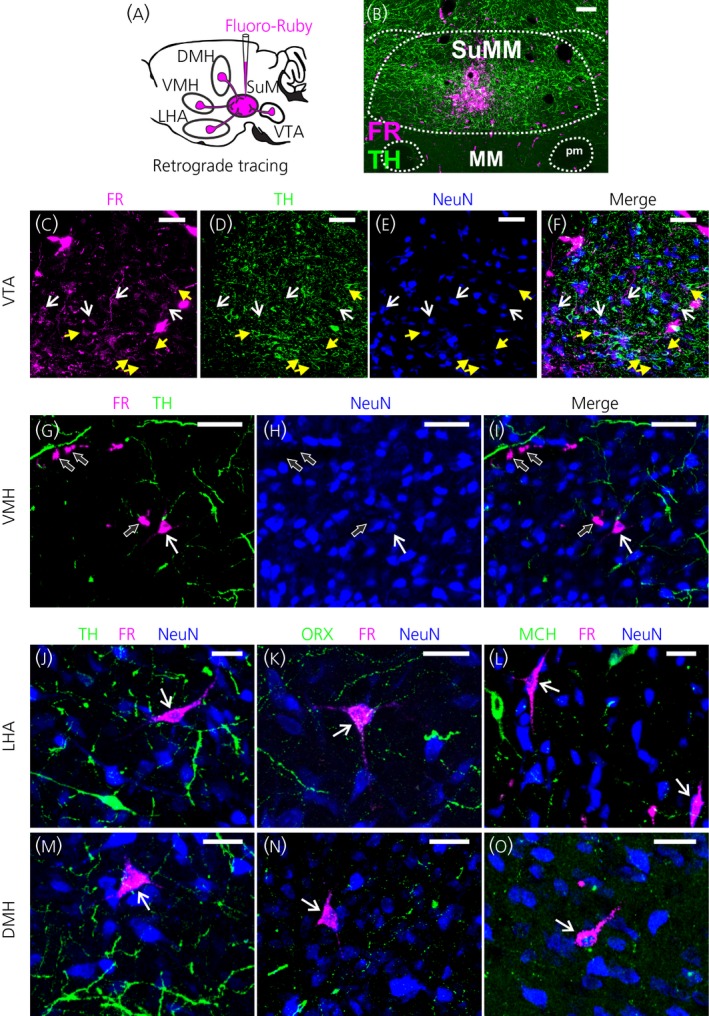
Retrograde labelling from the medial supramammillary nucleus (SuMM) in appetite and reward related areas. A, Schematic of the experimental procedure. Injection of Fluoro‐Ruby (FR) into the SuMM results in a fluorescent signal in regions that project to the SuMM. B, Representative image showing the iontophoretic injection site of FR confined to the SuMM at bregma −4.5 mm. (C‐O) Representative images of FR+ retrogradely labelled neurones (NeuN+) in the rostral part of the ventral tegmental area (VTAR) (C‐F; bregma −5.04 mm; yellow arrowheads point to neurones triple‐labelled for FR, tyrosine hydroxylase (TH) and a neurone‐specific marker (NeuN) and white arrows point to TH‐negative FR+ neurones), the ventromedial nucleus (VMH) (G‐I; bregma −2.52 mm; white arrows point to FR‐labelled neurones that were TH‐negative and black arrows point to nonspecific FR labelling), the lateral hypothalamic area (LHA) (J‐L; bregma −1.56 mm [J] and −2.52 mm [K, L]; white arrows point to FR‐labelled neurones that were TH‐negative, ORX‐negative or MCH‐negative) and the dorsomedial nucleus of the hypothalamus (DMH) (M‐O; bregma −2.76 mm [M] and −3.12 mm [N, O]; white arrows point to FR‐labelled neurones that were TH‐negative, ORX‐negative or MCH‐negative). Scale bars represent 200 µm (B), 100 µm (C, D), 50 µm (E‐I) and 25 µm (J‐O). ARC, arcuate nucleus; fr, fasciculus retroflexus; MM, medial mammillary nucleus; PBP, parabrachial pigmented nucleus; pm, principal mammillary tract; SUM, supramammillary nucleus; VTA, ventral tegmental area

In total, five injections of FR were analysed. These were selected because injection sites were centred in the rostro‐caudal axis of the SuMM between the two principal mammillary tracts with minimal spread into adjacent areas, and tracer labelling was not detected in the medial mammillary nucleus (Figure 2A,B).

Many retrogradely neurones (two to five per section) were found in the rostral part of the VTA along its rostro‐caudal axis from bregma −4.68 to −5.04 mm (Figure 2C‐F). The LHA harboured the greatest number of retrogradely labelled neurones (two to five per section) from bregma −1.56 to −3.6 mm (Figure 2J‐L). Fewer labelled neurones (from none to two per section) were found in the VMH from bregma −1.8 to −3.24 mm (Figure 2G‐I) and in the DMH from bregma −2.52 to −3.48 mm (Figure 2M‐O). We also observed retrogradely labelled neurones in the lateral septal nucleus (LS) from bregma +1.56 to +0.48 mm, especially at the border with the medial septal nucleus (Figure 4V‐Y). No labelled cells were seen in the cingulate cortex or arcuate nucleus.

To determine the neurochemical nature of the inputs to the SuMM, we co‐immunostained the FR‐labelled sections for TH in the rostral part of the VTA (VTAR) and VMH, or for TH/ORX/MCH in the LHA and DMH. We did not observe a colocalisation of ORX or MCH or TH with the retrogradely labelled neurones in the LHA or DMH. Similarly, no colocalisation with TH was observed in the VMH. In the VTAR, we observed retrogradely labelled neurones that were either immunopositive or immunonegative for TH (Figure 2C‐F).

### 3.3 The LHA and DMH receive input from the SuMM

**Figure 3:**
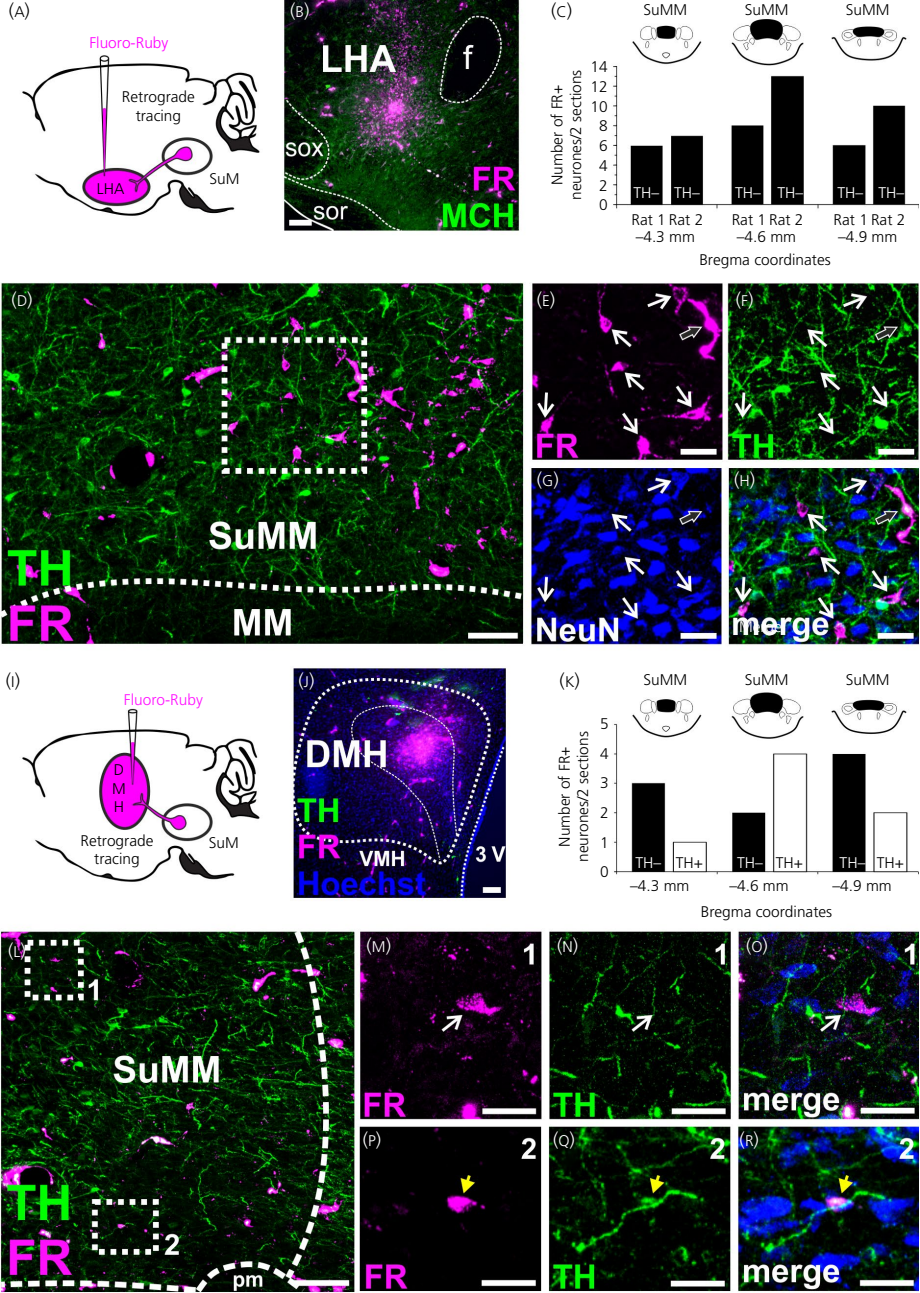
Retrograde labelling from the lateral hypothalamic area (LHA) and dorsomedial nucleus of the hypothalamus (DMH) in the medial supramammillary nucleus (SuMM). A, Scheme of the experimental procedure. Injection of Fluoro‐Ruby (FR) into the LHA results in a fluorescent signal in SuMM cells that project to the LHA. B, Representative image showing the iontophoretic injection site FR in the LHA at 2.4 mm posterior to bregma. Double‐labelling immunofluorescence for a neurone‐specific marker (NeuN) and tyrosine hydroxylase (TH) was performed to count and determine the chemical nature of the retrogradely labelled (FR+) neurones by confocal microscopy. C, Distribution of retrogradely labelled TH‐negative SuMM neurones (n = 2). D‐H, Representative confocal image of retrogradely labelled neurones in the SuMM taken at 4.5 mm posterior to bregma. The region in the white box in (D) is shown at higher magnification in (E‐H). To identify the labelled cells, TH, FR and NeuN‐labelling of the same cells are shown in separate images (E‐H). The white arrows indicate examples of FR‐labelled neurones that were TH‐negative. The black arrow indicates nonspecific labelling that can be observed following injection of FR. I, Scheme of the experimental procedure. Injection of FR into the DMH results in a fluorescent signal in SuMM cells that project to the DMH. J, Representative image showing the iontophoretic injection site of FR in the DMH at 3 mm posterior to bregma. K, Distribution of retrogradely labelled TH+ and TH‐negative SuMM neurones (n = 1). L‐R, Representative confocal images of retrogradely labelled SuMM neurones at 4.5 mm posterior to bregma. Regions in the white boxes 1 and 2 in (L) are shown at higher magnification in (M‐O) and (P‐R), respectively. (M‐R) To facilitate identification of the labelled cells, TH, FR and NeuN‐labelling of the same cells is shown in separate images. The white arrows indicate one example of FR‐labelled neurone that was TH‐negative (M‐O), the yellow arrowhead indicate one example of FR‐labelled neurone that were TH+ (P‐R). Scale bars represent 100 µm in (B, D, J, L) and 25 µm in (E‐H, M‐R). 3V, third ventricle; f, fornix; pm, principal mammillary tract; MM, medial mammillary nucleus; sor, supraoptic nucleus retrochiasmatic part; sox, supraoptic decussation; SUM, supramammillary nucleus; VMH, ventromedial nucleus

Our description is based on the analysis of two injections in LHA and one injection in DMH. Injection sites were restricted to the LHA and DMH (Figure 3B,J). Retrogradely labelled neurones were observed in the SuMM along its rostro‐caudal axis. We counted three rostral‐caudal levels of the SuMM (Figure 3C,K). Compared to the DMH, the LHA received more neuronal inputs from the SuMM. The highest number of SuMM neurones retrogradely labelled from the LHA was found at bregma −4.6 mm (13 cells per two sections analysed). Fewer labelled neurones were found in the SuMM when the FR was injected in the DMH (four cells at most per two sections analysed). To determine the neurochemical nature of these two inputs, the number of SuMM TH‐positive retrogradely labelled neurones was quantified. All the retrogradely labelled neurones were immunonegative for TH following injection in the LHA (Figure 3C), whereas seven retrogradely labelled neurones from the three rostral‐caudal levels were TH‐positive following the injection in the DMH (Figure 3K).

### 3.4 AAV‐mediated anterograde tracing

To confirm the existence of a reciprocal connection between the SuMM and the DMH, we used a viral labelling approach by injecting a Cre‐dependent virus into the SuMM or DMH of TH‐Cre rats. We performed serial injections (n = 12 in SuMM, n = 12 in DMH) with decreasing volume of AAV (1 µL to 100 nL). We selected injections that showed the optimal combination of spatial specificity of the injection site and a sufficient viral expression to evaluate the anterograde labelling to the projection areas. The animals selected in which the Cre‐dependent virus was injected into the SuMM (n = 1) and DMH (n = 1) involved an injection volume of 500 and 250 nL, respectively.

**Figure 4:**
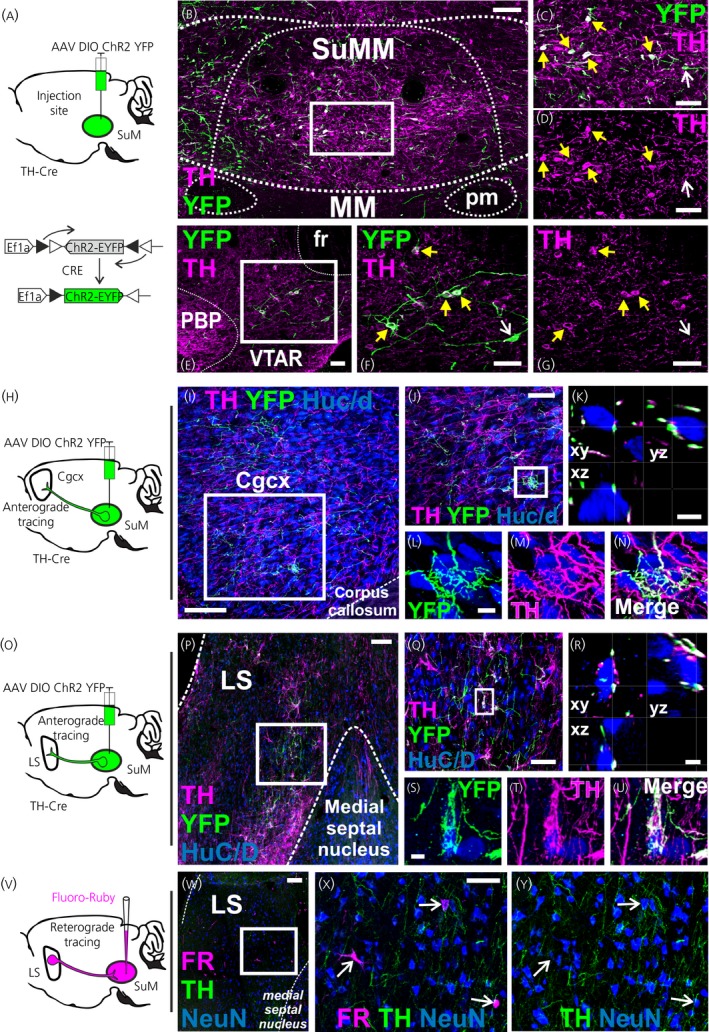
Connections between the medial supramammillary nucleus (SuMM) and the ventral tegmental area (VTA), lateral septal nucleus and cingulate cortex. A, Scheme of the experimental procedure. Injection of a DIO‐ChR2‐yellow fluorescent protein (YFP) adenoassociated virus (AAV) results in YFP expression in the SuMM. B‐D, Representative confocal images showing the injection site of DIO‐ChR2‐YFP AAV in the SuMM of rats expressing Cre recombinase under the tyrosine hydroxylase (TH) promoter (TH‐Cre) rats (4.5 mm posterior to bregma). E‐G, YFP expression in cell bodies of neurones in the rostral VTA (5.04 mm posterior to bregma). Regions in the white boxes in (B) and (E) are shown at higher magnification in (C, D) and (F, G), respectively. Yellow arrowheads indicate examples of YFP expression in cell bodies of TH+ neurones. White arrows indicate example of YFP expression in cell bodies of TH‐negative neurones. H and O, Schemes of the experimental procedure. Injection of DIO‐ChR2‐YFP AAV in the SuMM results in YFP‐labelled fibres in the cingulate cortex and lateral septal nucleus. Representative confocal images showing the AAV‐mediated anterograde labelling observed in the cingulate cortex (I‐K) and lateral septal nucleus (P‐R) at 0.48 mm anterior to bregma following the injection in the SuMM. Confocal photomicrographs (flattened stack of 30 optical sections at intervals of 1 μmol L^‐1^) illustrate neurones contacted by YFP+/TH+ labelled fibres in the cingulate cortex (L‐N) and lateral septal nucleus (S‐U). K and R, Contacts were confirmed in 3D with single optical sections in the *xy*‐, *xz*‐ and *yz*‐plane. V, Scheme of the experimental procedure. Injection of Fluoro‐Ruby (FR) into the SuMM resulted in a fluorescent signal in the lateral septal nucleus. W, Representative confocal images showing FR retrogradely labelled neurones in the lateral septal nucleus (0.48 mm anterior to bregma). The region in the white box in (W) is shown in higher magnification in (X, Y). The white arrows indicate three examples of FR+ retrogradely labelled neurones (NeuN+) that were TH‐negative. Scale bars represent 100 µm (B, I, P, W), 50 µm (C‐G, J, Q, X, Y) and 5 µm (K, L‐N, R, S‐U). Cgcx, cingulate cortex; LS, lateral septal nucleus; MM, medial mammillary nucleus; SUM, supramammillary nucleus; VTAR, ventral tegmental area (rostral part)

In the SuMM, TH‐immunostaining of brain sections at different levels of the rostro‐caudal axis revealed that the majority of ChR2‐YFP expressing neurones were TH‐positive (eYFP+/TH‐positive: 80.9 ± 2.7%; eYFP+/TH‐negative: 18.0% ± 2.7%; n = 3 rats) (Figure 4B‐D). We observed the expression of ChR2‐YFP in the rostral part of the VTA (5.04 mm posterior to bregma) (Figure 4E‐G), probably because the rostral part of the VTA is immediately adjacent to SuMM. Confocal microscopy revealed abundant TH‐positive projections to two extra‐hypothalamic areas previously described being connected with the SuM^17^: the LS and cingulate cortex. YFP+/TH‐positive fibres were observed making appositions with neuronal cell bodies in these regions, confirming successful delivery of the Cre‐dependent virus (Figure 4H‐U).

**Figure 5:**
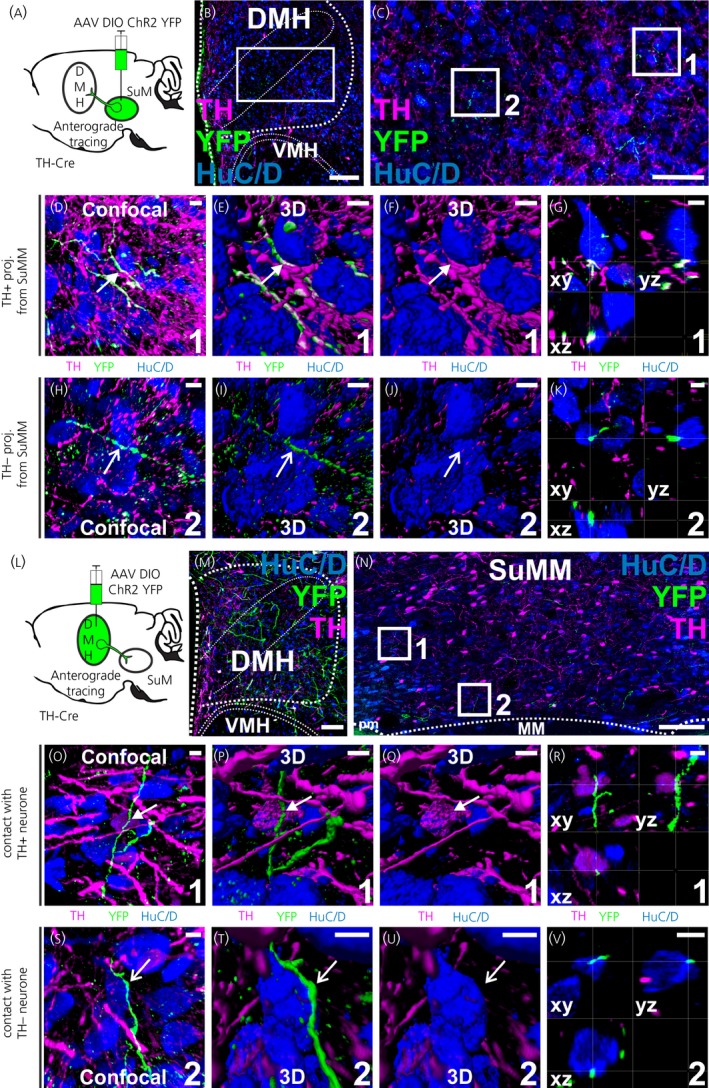
Reciprocal connections between the medial supramammillary nucleus (SuMM) and the dorsomedial nucleus of the hypothalamus (DMH). A, Scheme of the experimental procedure. Injection of DIO‐ChR2‐yellow fluorescent protein (YFP) adenoassociated virus (AAV) in the SuMM results in YFP‐labelled fibres in the DMH. B and C, Representative confocal images showing the AAV‐mediated anterograde labelling in the DMH (3 mm posterior to bregma). YFP‐labelled fibres in the white box in (B) are shown at higher magnification in (C). Regions in the white boxes 1 and 2 in (C) are shown at higher magnification in (D‐F) and (H‐J), respectively. D‐K, Confocal photomicrograph (D, H; flattened stack of 30 optical sections at intervals of 1 μmol L^‐1^) and 3D reconstructions (E‐G and I‐K) illustrate two examples of DMH neurones contacted by YFP+/TH+ (D‐G) and YFP+/TH‐negative labelled fibres (H‐K). G, K, All close appositions are confirmed in 3D with single optical sections in the *xy*‐, *xz*‐ and *yz*‐plane. L, Scheme of the experimental procedure. Injection of DIO‐ChR2‐YFP AAV in the DMH results in YFP‐labelled fibres in the SuMM. M, Representative confocal images showing the injection site of DIO‐ChR2‐YFP AAV in the DMH (3 mm posterior to bregma). N, YFP labelling was observed in the SuMM (4.5 mm posterior to bregma). Regions in the white boxes 1 and 2 in (N) are shown at higher magnification in (O‐Q) and (S‐U), respectively. (O‐V) Confocal photomicrographs (O, S; flattened stack of 30 optical sections at intervals of 1 μmol L^‐1^) and 3D reconstructions (P‐R and T‐V) illustrate examples of SUMM TH+ (O‐R) and SUMM TH‐negative neurones (S‐V) observed with YFP+‐TH‐negative appositions (arrows). R, V, All close appositions are confirmed in 3D with single optical sections in the *xy*‐, *xz*‐ and *yz*‐plane. Scale bars represent 100 µm (B, M), 50 µm (C) and 5 µm in all other images. MM, medial mammillary nucleus; pm, principal mammillary tract; SUM, supramammillary nucleus; VMH, ventromedial nucleus; VTA, ventral tegmental area

In the DMH, we found YFP+/TH‐positive and YFP+/TH‐negative fibres forming appositions with neurones (Figure 5A‐K). Only a few YFP+/TH‐negative expressing fibres were observed in the SuMM following injection of the AAV in DMH (Figure 5M‐V). The identified YFP+ fibres were found in close association with both TH‐positive and TH‐negative neurones. All appositions were verified in confocal microscope images in three planes to ensure that they did not represent superimpositions (Figure 5G,K,R,V).

## 4 DISCUSSION

Currently, the majority of research on the SuM focuses on its role in generating and influencing hippocampal theta rhythm via its projections to the hippocampus.^18^, ^19^, ^20^ However, recent findings provide evidence indicating that the SuM may be involved in metabolic control. We previously showed that the activity of SuM neurones is sensitive to ghrelin administration or to physiological states where ghrelin levels are elevated, and that intra‐SuM ghrelin administration promotes feeding.^1^ This novel role for the SuM in ghrelin‐associated feeding behaviours raises the question of whether the SuM is a node in the networks involved in motivation and appetite. Classical tracing studies have shown that the SuM projects to a number of brain regions.^6^ However, until now, the connectivity of the SuM with appetite‐ and motivation‐associated brain regions has not been directly investigated.

**Figure 6:**
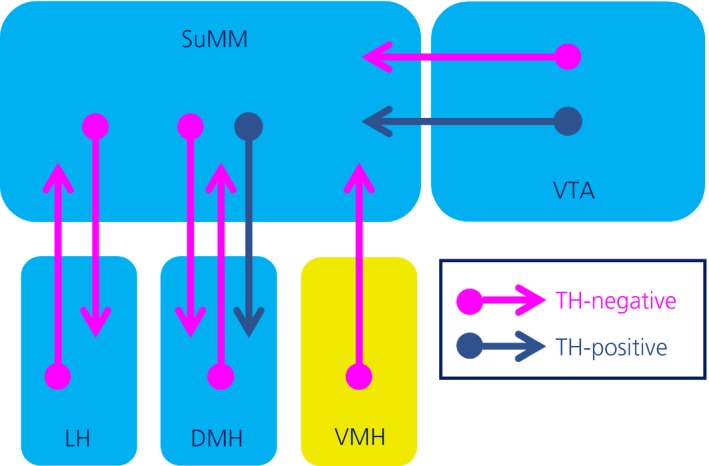
Depiction of tyrosine hydroxylase (TH)‐positive and TH‐negative connections between the medial supramammillary nucleus (SuMM) and selected appetite/reward areas. Regions in blue show increased levels of Fos expression after consumption of sweetened condensed milk (SCM), the region shown in yellow show no change after consumption of SCM. DMH, dorsomedial nucleus of the hypothalamus; LHA, lateral hypothalamic area; SuM, supramammillary nucleus; VMH, ventromedial nucleus; VTA, ventral tegmental area

We used Fos immunohistochemistry to indirectly assess neuronal activity in the SuM and other appetite‐ and motivation‐associated brain regions following the consumption of a high‐sugar, high‐fat food. Our data show that SuMM neurones, including those expressing TH, are activated following the motivated consumption of this food by satiated rats, and this activity is positively correlated with the activity of neurones in the LHA, DMH and VTA (summarised in Figure 6). This suggests that these nuclei may display functional connectivity stimulated by the consumption of an energy‐dense food.

Interestingly, we observed an increase in Fos expression in the SuMM after rats were given access to SCM for the first time (“novel” access). This increase was observed in cells that were not TH‐positive, although it occurred regardless of whether the rat consumed the SCM. It is difficult to interpret this observation, although it may point to a role for the SuMM in learning and/or the physiological response in food neophobia.^21^

To determine whether Fos expression was related to anticipation of SCM access, we also measured Fos expression in rats that were schedule‐fed SCM, and then were presented with SCM several hours earlier than the anticipated time. Fos expression was increased in the SuMM, although there was no difference in Fos expression in rats that received SCM at the expected or unexpected time. All rats ate the SCM in this experiment, suggesting that this increase in Fos expression is a result of the consumption of SCM, and not anticipation of access to SCM (nor an effect potentially related to neophobia). By contrast to novel access, where Fos expression did not increase in TH‐positive cells, scheduled access did result in an increase in Fos expression in TH‐positive cells, pointing to a role for TH‐positive cells in the drive to consume or in the physiological consequences of consumption.

The mechanism underlying these observations is not known, although rats compensate for SCM consumption by reducing their intake of standard diet,^16^ suggesting that the difference seen here are not a consequence of a change in body weight or an increase in total daily energy intake. The hypothalamic neurohormone oxytocin has an emerging role in appetite control, and may be involved specifically in an anorexigenic response to carbohydrate intake.^22^ Oxytocin may have a neuromodulatory role in the SuM,^23^, ^24^ and we have previously shown that gavage of a high‐sugar food increases the electrical activity of oxytocin neurones in the rat supraoptic nucleus,^9^ which may lead to somatodendritic oxytocin release to target the SuM. Alternatively or additionally, ghrelin signalling may be relevant. Ghrelin is orexigenic when administered into the brain, and increases in ghrelin levels are also associated with anticipation of palatable schedule‐fed food in rats.^25^ We have previously shown that rat SuM neurones are sensitive to this hormone.^1^ Similarly, GLP‐1 levels increase in the rat before food access in a model of scheduled feeding of a standard diet,^26^ and motivation‐related brain nuclei are sensitive to analogues of this hormone when administered centrally.^3^

We used retrograde and anterograde tracers, and Cre‐dependent viral‐assisted mapping, to trace the afferent and efferent connections of SuMM neurones. We identified direct connections between the SuMM and the LHA, DMH and VTA; areas where Fos expression correlated with that seen in the SuM. The connection between the SuMM and LHA and DMH appears to be reciprocal. We also confirmed an efferent connection from the VMH, a primary satiety centre in the hypothalamus, to the SuMM.^27^ The fact that the SuMM receives direct inputs from the LHA, DMH, VMH and VTA, as well as the anatomical position of the SuM in the hypothalamus, supports the idea that the SuM may act as a relay centre, integrating information from appetite and reward‐associated regions in the regulation of motivated feeding behaviours.

The VTA is a well‐characterised node in brain reward circuitry. VTA neurones, notably those releasing dopamine, project to multiple brain regions.^28^ In the present study we provide evidence indicating that the SuMM is a target of both TH‐positive (presumably dopaminergic) and TH‐negative VTA neurones. This aligns with the well‐established functional roles of the VTA and the proposed role for the SuM in reinforcement of motivated behaviour.^4^, ^29^ However, the identity of these VTA inputs, their targets in the SuMM and the functional relevance of these connections all remain to be determined. Similarly, the LS is involved in food‐directed motivation,^30^ and we show a direct projection from the SUMM to LS, providing further evidence for the involvement of the SuM in motivated behaviour.

We also provide evidence for a direct reciprocal interaction between the SuMM and the LHA and DMH. The LHA hosts a heterogeneous population of neurones and is considered to be an interface between the hypothalamus, the mesolimbic reward system and other areas involved in food‐motivated behaviour.^31^ LHA, MCH and ORX neurones function as sensors of the internal metabolic environment,^32^, ^33^, ^34^ and activation of orexin neurones (which project to dopaminergic neurones in the VTA) appears to link metabolic state and reward‐based feeding.^35^ For example, blockade of the ORX pathway blunts the effects of ghrelin on the reward system.^36^ In addition, there is evidence for a population of TH‐positive neurones in the LHA,^37^ although their function and connectivity are not known.^38^ Interestingly, in our study the LHA neurones projecting to the SuMM do not appear to express either ORX, MCH or TH.

It has been reported previously that ORX LHA neurones project to the SuM.^39^ However, we did not confirm this finding. This discrepancy could be explained by our methodological refinement of using small injection volumes. LHA ORX neurones also project to the VTA.^39^, ^40^ Therefore, it is possible that ORX fibres simply pass through the SuM before they terminate on VTA neurones. Alternatively, these LHA ORX neurones may only project to the SuML and not the SuMM. The identity of this LHA projection to the SuMM is unknown.

Recent evidence indicates that the DMH is an important orexigenic centre in the brain acting via leptin‐sensitive GABAergic outputs^41^ and neuropeptide Y receptor‐expressing neurones,^42^ or through inhibition of paraventricular nucleus of hypothalamus by GABAergic projections.^43^ To the best of our knowledge, the present study is the first to clearly show a projection from the DMH to the SuM, suggesting a reciprocal connection between these two nuclei. TH, ORX and MCH‐expressing neurones are also located in DMH^7^, ^44^, ^45^ but, according to our findings, these neurones do not project directly to the SuMM.

Previous retrograde^46^ and anterograde tracing studies have reported a direct projection from the SuMM to the DMH.^17^ We have identified both TH‐positive and TH‐negative SuMM neurones in this projection. The identity of these TH‐negative neurones is unknown, although the SuMM has been shown to contain a wide variety of different neuronal subtypes.^47^, ^48^, ^49^ TH‐positive SuMM neurones projecting to the DMH are potentially dopaminergic because they do not express the noradrenergic synthetic enzyme, dopamine‐b hydroxylase.^50^, ^51^ The axosomatic appositions revealed in the present study suggest synaptic contacts, although, without electron microscopic analysis, we were unable to confirm the synaptic nature of these appositions.

We provide evidence for a connection projecting from the VMH to the SuMM. Despite this, we did not observe a change in levels of Fos expression in the VMH after SCM consumption. However, we did not seek to determine which VMH neuronal subtypes were activated after consumption compared to cells activated in control rats, and it may be possible that different cell types are activated in these different conditions without a significant change in the total number of cells activated.

Interestingly, we did not observe direct connections between the SuMM and the arcuate nucleus, a region considered to contain the ‘first‐order’ neurones in the regulation of appetite and feeding behaviour.^52^ However, arcuate nucleus neurones connect to the LHA^53^ and DMH,^54^ suggesting that communication between the arcuate nucleus and the SuMM could occur indirectly, via these regions.

Despite our attempts to restrict the injection site to the SuMM, we observed viral YFP expression in neuronal cell bodies of the rostral part of the VTA, rendering the anterograde tracing results more difficult to interpret. Previous reports indicate that LS receives inputs from both the VTA and SuM, and this projection is known to be dopaminergic and cholecystokininergic.^50^, ^55^, ^56^ The contacts in the LS shown in the present study may correspond to the dense pericellular baskets of dopamine fibres seen around neurones.^57^ We observed a specific input from the LS to the SuM, confirming the bidirectional connection between the SuM and LS. This projection is located at the border between the lateral septum and medial septum and involves GABAergic projections to calretinin‐containing neurones in the SuM.^58^, ^59^

It is well documented that VTA project to cingulate cortex via the mesocortical dopamine pathway. Although this neurochemical phenotype remains unidentified, SuM projections to the cingulate cortex originate chiefly from the SuML.^17^, ^60^ Therefore, we consider the TH‐positive immunoreactive fibres in cingulate cortex described in the present study to represent dopaminergic axons arising from the VTA rather than the SuMM.

In our earlier electrophysiological study of the SuM,^1^ we did not determine the neuroanatomical connectivity of the cells we recorded in vivo. Approximately 40% of the cells we recorded were juxtacellularly labelled with neurobiotin but none of these neurobiotin‐labelled cells proved to be TH‐positive. As such, it is difficult to relate the electrophysiological characteristics of these recorded SuM cells with a potential role in the connectivity described here.

In the present study, we used small injection volumes to ensure localised transfection and labelling, adopted a viral‐based approach for specific identification and tracing of TH‐expressing cells, and made a rigorous assessment of putative axosomatic contacts. We demonstrate a reciprocal connection between the LHA and SuMM, adding substantially to a body of evidence for a reciprocal connection between DMH and SuMM. We show that ORX/MCH/TH‐negative DMH neurones innervate the SuMM, and also show the existence of a potentially dopaminergic SuM output to the DMH. Taken together, these data support the concept that the SuM is part of the brain's appetite control and/or motivational system^1^, ^2^, ^3^, ^4^, ^5^, ^29^, ^61^ and open avenues to further work characterising the functional circuitry involved in these behaviours.
