# Supplementary figures and images for: Neural connectivity between the hypothalamic supramammillary nucleus and appetite‐ and motivation‐related regions of the rat brain
Source: J Neuroendocrinol. 2020 Jan 29;32(2):e12829. doi: 10.1111/jne.12829 (PMC7065010; doi:10.1111/jne.12829)

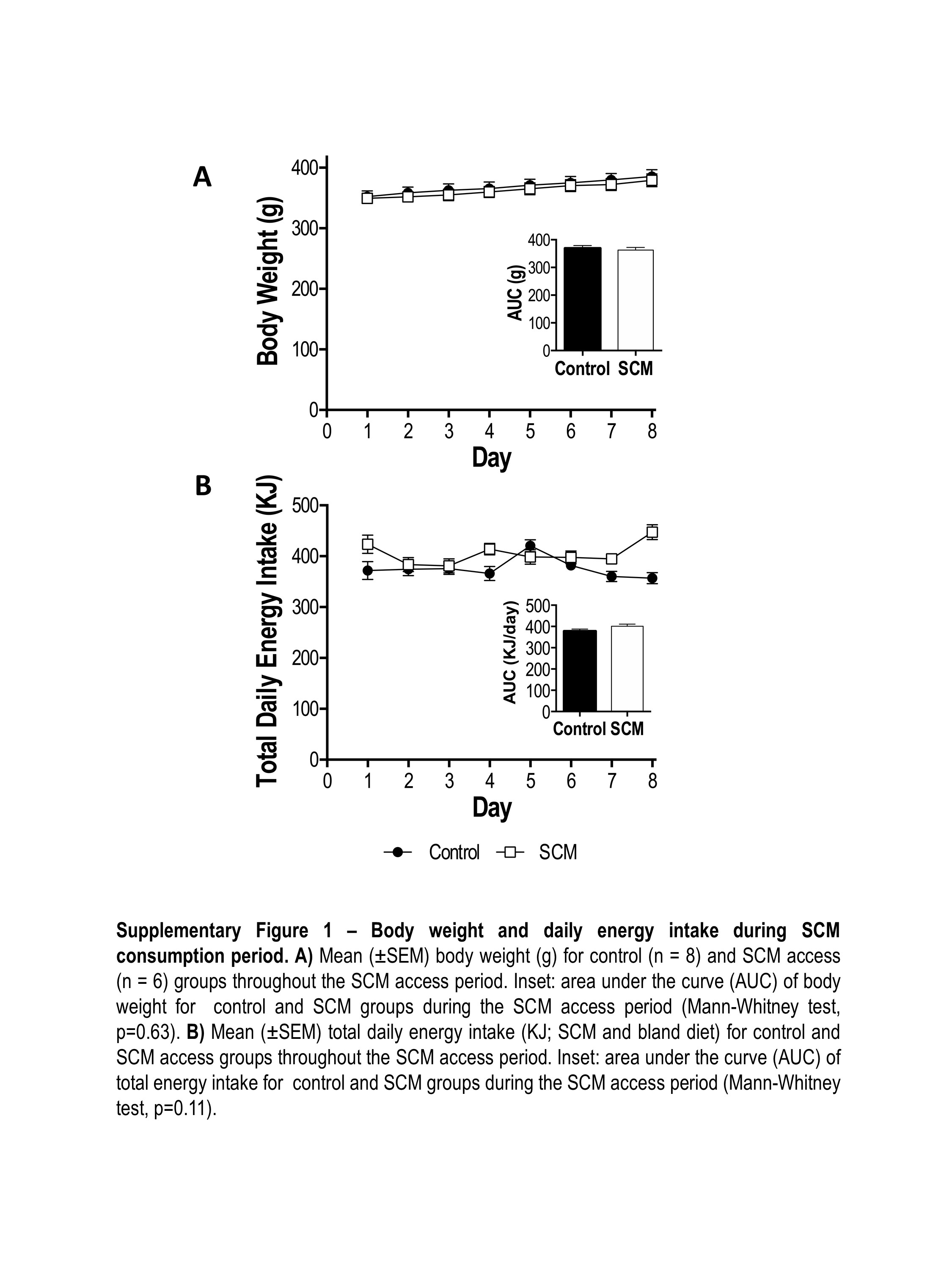

Supplement: Supplementary file 1 [file JNE-32-e12829-s001.jpg]
